# Anti-apoptotic Effects of Bone Marrow on Human Islets: A Preliminary Report

**DOI:** 10.4172/2157-7633.1000274

**Published:** 2015-04-09

**Authors:** Lu-Guang Luo, John ZQ Luo

**Affiliations:** 1 Roger Williams Medical Center, Boston University, USA; 2 Brown University, Alpert Medical School, Providence, Rhode Island, USA

**Keywords:** Bone marrow, Human islet, IL-1β, Mir146a, Anti-apoptosis

## Abstract

Apoptosis is one of the major factors contributing to the failure of human islet transplantation. Contributors to islet apoptosis exist in both the pre-transplantation and post transplantation stages. Factors include the islet isolation process, deterioration in vitro prior to transplantation, and immune rejection post transplantation. Previous studies have demonstrated that co-cultured bone marrow cells with human islets not only significantly enhanced the longevity of human islets but also maintained function. We hypothesized that the protective effects of bone marrow cells on human islets are through mechanisms related to preventing apoptosis. This study observed the levels of inflammatory factors such as interleukin-1β (IL-1β), the release of extracellular ATP in vitro, and expression levels of P2X7 ATP receptor (P2X7R), all of which lead to the occurrence of apoptosis in human islets. When human islets were co-cultured with human bone marrow, there was a reduction in the rate of apoptosis correlated with the reduction in inflammatory factors, extra cellular ATP accumulation, and ATP receptor P2X7R expression versus human islets cultured alone. These results suggest that co-culturing bone marrow cells with human islets inhibits inflammation and reduces apoptosis, thus protecting islets from self-deterioration.

## Introduction

Human pancreatic islet transplantation for the treatment of Type 1 diabetes has been hindered by challenges related to maintaining islet survival and function *in vivo*. Many factors contribute to islet damage both in the pre-transplantation and post-transplantation stages. It is estimated that up to 60% of pancreatic islet tissue undergoes apoptosis *in vitro* [1,2]. Harvesting human islets from the donor results in the loss of vasculature leading to β cell apoptosis due to their sensitivity to hypoxic conditions [3]. The apoptotic pathways in islet cells are triggered by the disruption of the islet cell matrix during the isolation process [4]. The storage of human islet following isolation is another challenge since the quality of human islets deteriorates rapidly [5]. A recent study discovered that when isolated human islets were cultured *in vitro* for more than three weeks, increased stress in the endoplasmic reticulum resulted in significant beta-cell apoptosis [6].

The current theory of cell apoptosis suggests two major pathways. In the first pathway, cells produce pro-apoptotic proteins in response to external stimuli such as mitochondria toxins and DNA damage. The pro-apoptotic proteins activate cytochrome C release from the mitochondria, which then activate caspase 9 and caspase-3, leading to cell apoptosis. The second type is inflammation related apoptosis, wherein cell receptors are activated by pre-inflammatory cytokines such as IL-1β, tumor necrosis factor-α (TNF-α), and interferon-γ (IFN-γ). The activation of these receptors caused by cytokines and other stressors induces the translocation of transcription factor nuclear factor-κB (NF-κB) from the cytosol into nucleus [7]. The relocation triggers the transcription of the NO synthase (iNOS) gene and the activation of the mitogen-activated protein kinases (MAPKs) which contribute to apoptosis [8]. Receptor activation could also trigger another path of cytokine-induced signaling which involves the activation of the mitogen-activated protein kinases (MAPKs) c-Jun NH2-terminal kinase (JNK), extracellular signal-regulated kinase (ERK), and p38 [9].

Post-transplantation apoptosis in human islets can be attributed by inflammatory factors. In the early stages of transplantation, human islet function is suppressed by pro-inflammatory cytokines [10,11], which can stimulate the c-JNK islet cell apoptosis pathway. Upon activation of the apoptosis pathway, human islet cells could consequently release more pro-inflammatory cytokines which could attract and activate leukocytes. Recruitment of activated leukocytes towards islets as a result of cytokine and chemokine secretion could induce islet injury and present foreign antigens to the memory/dendritic cells, which lead to an acute immune response, death or impairment of β cells. The specific cytokines, such as IL-1β, released by transplanted pancreatic islets may be directly related to islet survival after isolation and transplantation [12].

Isolated human islets have been shown to secrete TNF-α, IL-1β, IL-6, and nitric oxide. Secretion of these mediators was shown to be augmented by IL-1β [13] and other cytokines [14]. IL-1β is considered to be one of the key initiators of the inflammatory response. It is a pro-inflammatory cytokine that may mediate islet dysfunction and alter outcomes in the post-transplantation period [15]. IL-1β expression and release in human islet β cells has been observed in response to high glucose induced cell damage. The occurrence of cell death via the activation nuclear factor kappa B (NF-κB) and Fas apoptotic cascades leads to a suppression of islet function and destruction of β cells [13].

Preventing apoptosis through the inhibition of cytokine release or inhibition of proapoptotic cascades could help protect pancreatic islets from either cell-mediated or non-cell-mediated destruction [16]. For instance, ductal injection of JNK inhibitors was shown to prevent beta cell apoptosis and improve islet graft function [17]. Gene transfer of the insulin-like growth factor I (IGF-1), an inhibitor of IL-1β, into human islets reduced apoptosis by preventing IL-1β-induced β cell dysfunction and Fas-triggered apoptosis activation [18]. Supplementation of prolactin in the culture medium was also shown to protect human islet cells from cytokines, nitrix oxide, and H_2_O_2_, thus improving islet survival [19]. MSCs could protect islets against hypoxia/reoxygenation-induced injury by inducing hypoxia-resistant molecules, HIF-1α, HO-1, and COX-2 [20]. In another study, islets co-cultured with MSCs demonstrated lower ADP/ATP ratios and higher levels of anti-apoptotic signal molecules (X-linked inhibitor of apoptosis protein, Bcl-xL, Bcl-2, heat shock protein-32), higher levels of vascular endothelial growth factor receptor 2/Tie-2 mRNA expression, and increased levels of phosphorylated Tie-2 and focal adhesion kinase protein [21]. Co-culture of streptozotocin (STZ)-damaged rat pancreatic islets with bone marrow showed the expression of IL-6 and transforming growth factor-β1 in the culture medium in addition to some of the antiapoptotic genes (Mapkapk2, Tnip1 and Bcl3) [22], implying the cytoprotective, anti-inflammatory and antiapoptotic effects of BM are through paracrine actions.

Considering the species differences in human islets and rat islets in terms of sensitivity to cytokines [23], the aim of this study is to investigate the anti-apoptosis effect of human bone marrow cells on human islets and identify whether the corresponding mechanism is correlated in both human and animal tissue. In our previous study, we demonstrated that co-culturing bone marrow cells with human islets supported human islet survival and function for over six months [24]. In the co-culture system, we observed that bone marrow cells were able to both initiate angiogenesis and convert into insulin producing cells [25,26].

In the current study, we hypothesize that bone marrow cells suppress apoptosis in human islets and regulate inflammatory factors within the cell culture micro-environment. In order to test our hypothesis, we first investigated the apoptosis rate with and without bone marrow co-culture. The TdT-mediated dUTP nick-end labeling (TUNEL) method followed by immunohistochemistry staining was used to measure apoptosis. We then evaluated time-course changes in the expression of IL-1β, extracellular ATP release, the expression level of ATP receptor (P2X7R) expression within human islets in the co-culture system.

## Materials and Methods

### Human islet and bone marrow (BM) cell culture

#### Human pancreatic islets

Human islets, from healthy donors, were obtained from Integrated Islet Distribution Program (IIDP) in the IIDP Basic Science Islet Distribution Program, Human Islet Laboratory, University of Pennsylvania (Philadelphia, PA), Massachusetts General Hospital (Boston, MA) and City of Hope National Medical Center (Duarte, CA). The use of these cells is approved by the Institutional Review Board (IRB) at Roger Williams Hospital and the ICRs Committees.

#### Harvested human BM

Human BM from normal donors was obtained under a separate Roger Williams Hospital IRB approved protocol. Bone-marrow erythrocytes were isolated by Ficoll-Paque^™^ Plus (Amersham Biosciences; Amersham, UK) per manufacturer directions. Cells were then washed twice with 10% fetal calf serum (FCS) in phosphate buffered saline (PBS), re-suspended in culture medium (see below). Trypan blue staining was used (manufacturer) to assess for cell viability. Harvested human whole bone marrow included mesenchymal cells (>65%), endothelia cells (>30%) and other bone marrow stem cells (>5%) data published in other article.

#### Allogeneic BM co-cultured with human islets

Human islets were received from Islet Cell Resource Centers (ICRs) within 48 hours after harvest from normal/non diabetic cadaveric donors. The purity of islets in total isolated tissue was 75~90% as assessed by dithizone identification and viability was >95% as determined by trypan blue dye exclusion. Islets were placed in culture at 100 islet equivalents (IEQs) per ml with 1 × 10^6^ allogeneic BM cells/ml. Cultures were maintained in RPMI 1640 (GIBCO) supplemented with 10% heated inactivated fetal bovine serum (HiFBS, GIBCO), 5.5 mM glucose, 10 mM HEPES, and 1% P/S. Trypan blue staining (GIBCO) was used to identify the dead cells in human islet with/o bone marrow cells.

#### Labeling BM cells with PKH26

BM cells labeling with PKH26, a fluorescent membrane dye, was performed according to the manufacturer’s instructions (Sigma, St Louis, Mo.). Specifically, bone marrow cells (1 × 10^6^ allogeneic BM cells/ml) in culture medium were incubated with 0.2 μM PKH26 for 2 mins at room temperature. Cell images were taken under fluorescent microscopy.

#### Apoptosis detection via TUNEL staining

The detection of apoptotic nuclei using the TUNEL staining was performed with ApoAlert^™^ DNA Fragmentation Assay Kit (Clonetech, Palo Alto, CA). Briefly, cells were fixed in 4% paraformaldehyde and incubated in 0.3% H_2_O_2_ in methanol for five minutes to block endogenous peroxidase. The slides were then incubated with fluorescent-conjugated avidin in PBS for two hours at room temperature. After three washes, slides were cover slipped with glycerol phosphate buffer. Apoptotic cells were counted in at least 10 random fields and expressed as a percentage of the total cell number (apoptotic index).

#### Evaluation of human islet function

Islet function was evaluated by measurement of insulin release with or without glucose challenge. High-glucose challenge was performed according to the procedure described above. Insulin concentrations in the specimens (cell culture media or animal blood) were measured using a Human Insulin ELISA Kit (Milipore, Billerica, MA) according to the manufacturer’s instructions, and insulin concentrations were calculated using KC Junior microplate reader software (Bio-Tek Instruments, Inc.).

#### Extracellular ATP levels and IL-1β content in both human islet culture systems

Extracellular ATP levels, an indicator for human islet cell death, was evaluated under normal culture conditions as well as the high-glucose challenge conditions. High-glucose challenge was performed once a week as follows: media was collected and cultured cells were washed once with RPMI medium. Media was then replaced with high-glucose (20 mM) RPMI 1640 for 30 min. The media was then collected and stored at −80°C for analysis of extracellular ATP levels and IL-1β content. ATP levels in cell culture medium were analyzed with the ENLITEN^®^ ATP Assay System Bioluminescence Detection Kit (Promega) according to the protocol, and luminescence was measured on a Fluostar Optima instrument (BMG Labtechnologies, Germany). IL-1β content was analyzed with the BD OptEIA human IL-1β enzyme-linked immunosorbent assay (ELISA) kit II according to the protocol (BD Biosciences Pharmingen, San Diego, CA). IL-1β was determined by measuring the optical density at 450 nm in a Labsystem Multiscan Plus fluorescence spectrophotometer. IL-1β and insulin concentration tested on the same time point were correlated in order to identify the relation between these two parameters.

#### Fluorescent immunohistochemistry (FIHC)

P2X7 receptor expression in islet with or without co-culture was evaluated by double-labeled fluorescence immunohistochemistry (FIHC) using specific antibody for P2X7 receptor (LS-A9585, LSBio Inc. Seattle WA) and proinsulin (ab76570 ABcam, Cambridge, MA). FIHC procedures are as follows: cells grown on chamber slides were fixed with 4% paraformaldehyde, followed by exposure to 10% normal goat serum. The slides were blotted without washing; primary antibodies and a second fluorescent color and/or third antigen-detecting antibody were applied. When the process was finished, the slide was cover-slipped using a fluorescent mount medium. The samples were evaluated and photographed using confocal fluorescent microscopy.

The P2X7R antagonist oxidized ATP was applied to the human islet culture medium in order to verify whether the expression of P2X7R could be suppressed by adding the oxidized ATP. The expression level of P2X7R after one week treatment was examined by q-PCR analysis.

#### Statistical analysis

Data is presented as mean ± standard error (STD). Experiments were repeated three times from different donators unless otherwise noted. For figures and tables, the results from one experiment are shown. Data used for graphical presentation and statistical analysis are expressed as per experiment. Data sets were analyzed by ANOVA statistics program using a two factor analysis of variance of repeated measures. Post hoc comparisons among individual means were evaluated using a T-Test.

## Results

### Deterioration of human islet *in vitro*

**Figure 1:**
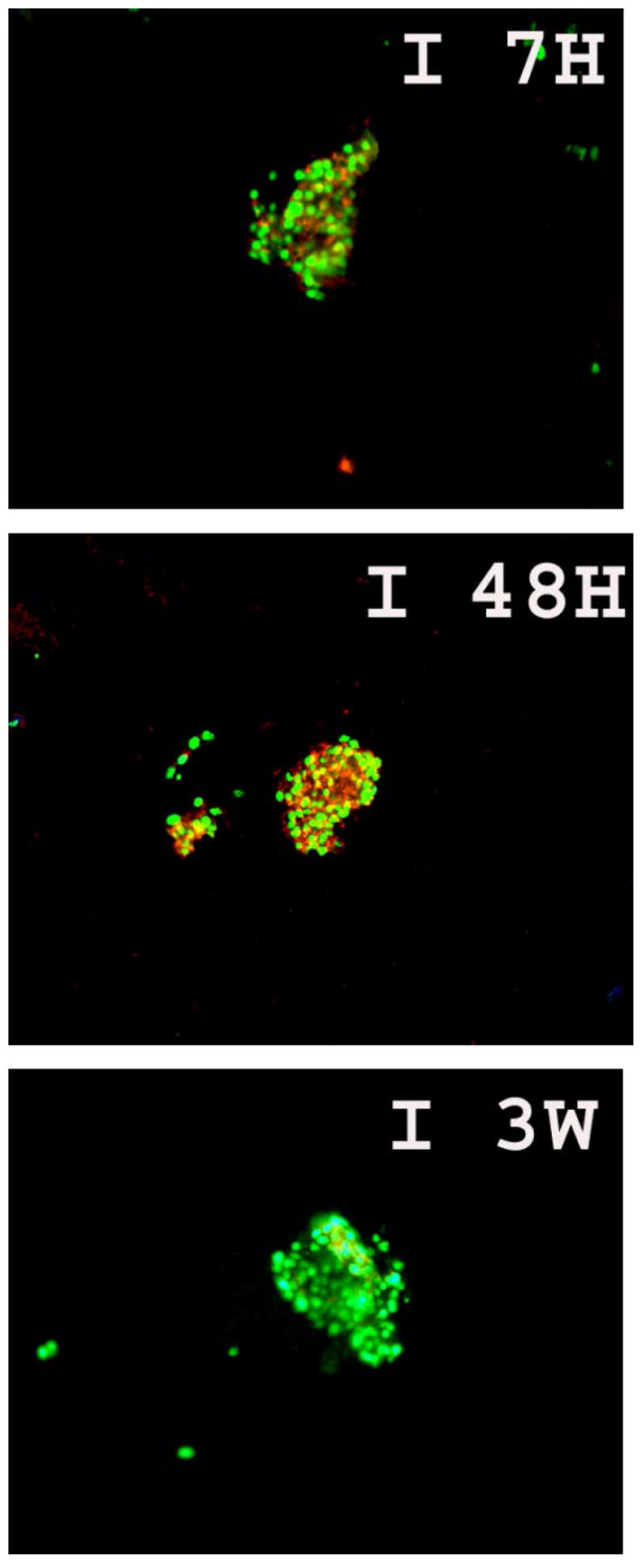
Levels of apoptosis in human islets cultured for 7 hours (A), 48 hours (B), and 3 weeks (C) evaluated by TUNEL assay (green) following pro-insulin fluorescent (red) immunohistochemistry to identify islet β cells.

**Figure 2:**
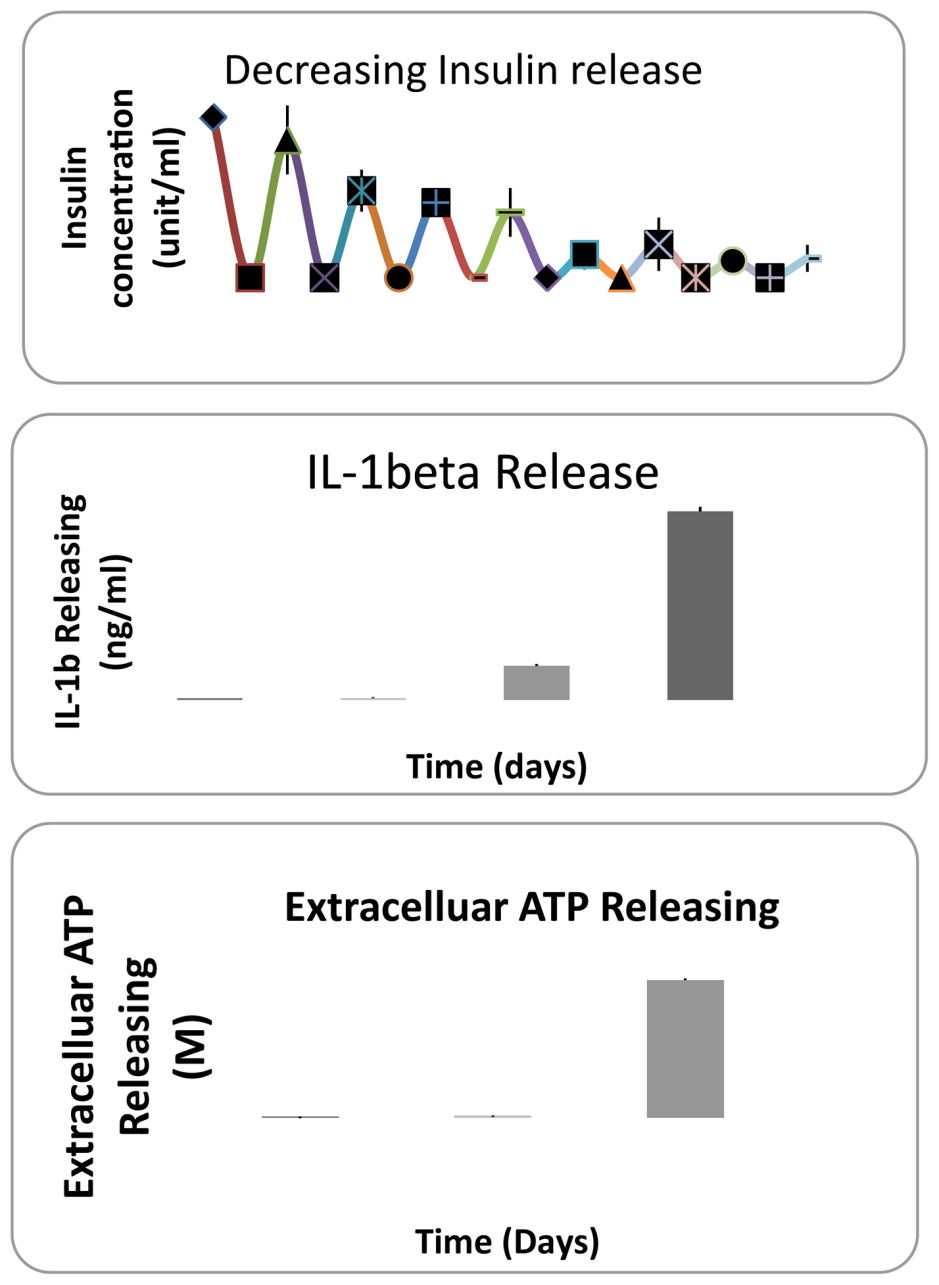
Decrease in human islet function in vitro. Measurement of insulin release on days 17, 20, 24, 27, 32, 34 and day 38 (A), IL-1β release on days 8, 15, 35 and 63 (B) and Extracellular ATP levels on day 8, 35 and 63(C).

Apoptosis in isolated human islets was observed after 7 hours, 48 hours and 3 weeks culture at 37°C as determined by TUNEL assay (Figure 1). We evaluated the human islet function by measuring the insulin release at day10, 14, 17, 20, 24,27,32,34, and 38. As shown in Figure 2A, insulin release declined to a base level after 38 days of culture.

### Enhanced inflammation in human islet in culture

In order to evaluate the inflammation level within human islet in culture, the concentration of IL-1β and ATP in the cultured medium were measured. It was found that both IL-1β release and extracellular ATP levels increased with the length of culture time (Figures 2B and 2C).

### BM co-culture maintained human islet morphology and viability

**Figure 3:**
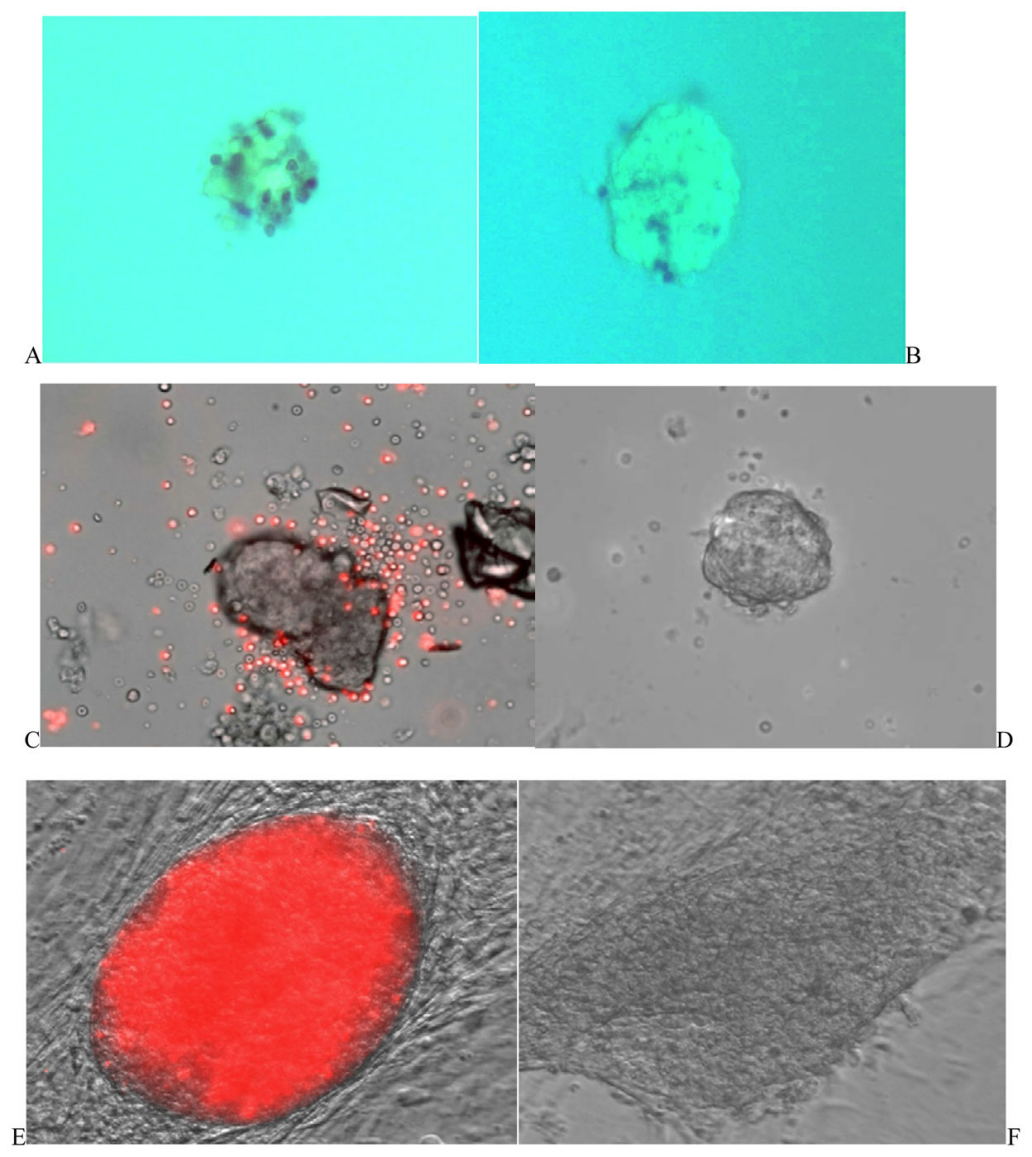
Bone Marrow improved islet viability (A, B) and maintained islet tissue structure (C–F). A, B: Light microscopy images of human islet only culture (A) and islet with BM co-culture (B) for three weeks. Dead cells were stained with trypan blue. C–F: Cell images observed under light microscopy and fluorescent microscopy. Human islets were co-cultured with Bone marrow cells (labeled with PKH26 in red color) in Petri dishes day 1 (C) and day 60 (E). Human islet only cultures in Petri dish on day 1 (D) and day 60 (F). Magnification: 40X.

In Figure 3A, human islets co-cultured with bone marrow were stained with trypan blue to indicate cell death. Results showed a significant improvement in islet viability in BM/islets co-cultured groups in comparison with islet only groups. The interaction of bone marrow cells and human islets in the co-culture system were observed under fluorescent microscope, wherein bone marrow cells were stained with PKH26 in red (Figures 3C–3F). It was found that without the support of bone marrow, human islets gradually lost structure and became monolayer on day 21 (Figure 3F). Co-cultured human islets continuously recruited bone marrow cells and formed cohesive structures (Figure 3E).

### BM co-culture reduces cell apoptosis in human islet

**Figure 4:**
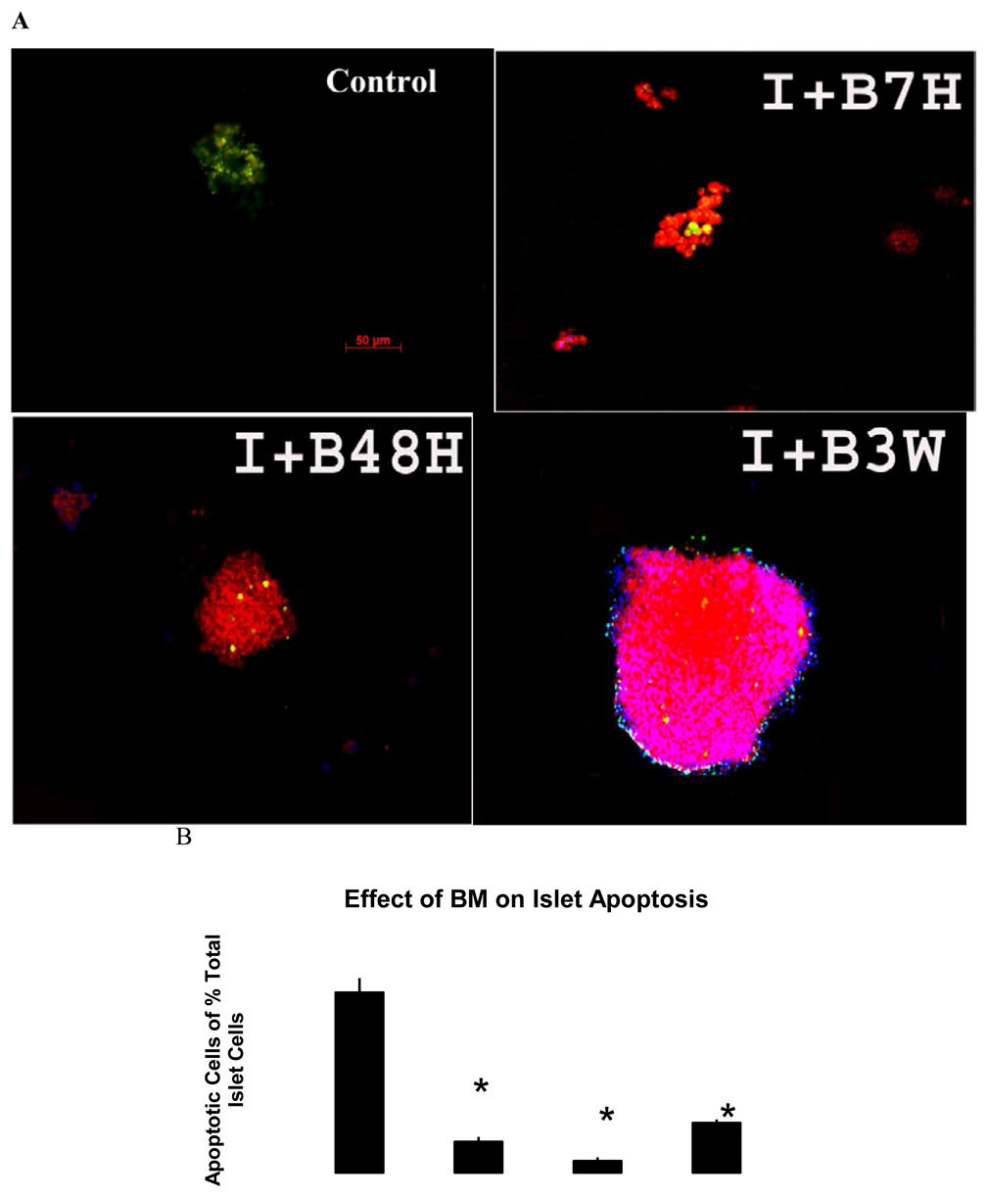
Islets cultured with BM for 7 hours, 48 hours, and 3 weeks and islet only culture for 7 hours as control were measured by TUNEL assay (green) for apoptosis following pro-insulin fluorescent (red) immunohistochemistry to identify islet β cells. The images on the top left indicate that islets co-cultured with BM (bottom row) have fewer apoptotic cells and stronger insulin staining than those in islet-only culture (top row) (I: Islets Only; I+B: Islet Co-cultured with BM; xH: Culture Hours; xW: Culture Weeks). b. Quantification of apoptotic cells as a percentage of total islet β cells in co-culture groups and human islet culture only groups for 7 hours, 48 hours and 3 weeks. *p<0.01vs. islet only culture (I7 h and I3 W).

An apoptosis detection kit (TUNEL ASSAY) was used to further evaluate apoptosis in β cells. Bone marrow co-culture was able to prevent β cell apoptosis. A reduction in apoptosis was observed in the co-culture group (Figure 4). In situ imaging results showed that BM co-culture increased insulin positive cells as well as significantly reduced the occurrence of cell apoptosis.

### BM increases pancreatic β Cell insulin secretion and reduces inflammatory factor IL-1β release in co-culture system

**Figure 5:**
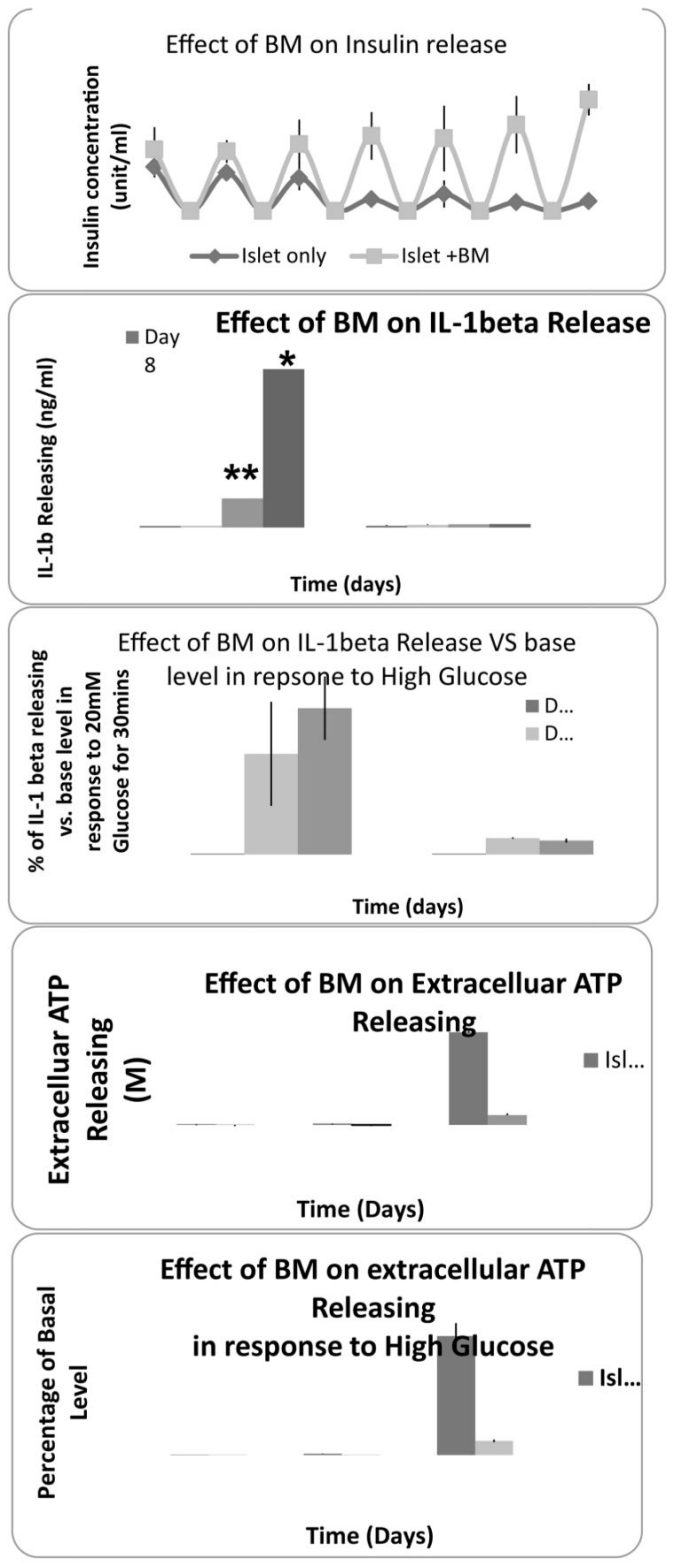
Effect of bone marrow cells co-culture on the function of human islets: (A) Insulin release; (B) IL-1beta release; (C) IL-1beta release in response to high-glucose challenge. (D) Extracellular ATP levels; (E) Extracellular ATP levels in response to high-glucose challenge.

Effects of BM co-culture were observed through increased insulin release levels in comparison with the control group (Figure 5A). The co-culture of bone marrow cells with human islets significantly suppressed the production of IL-1β not only under basal culture conditions (5.5 mM glucose concentration) but also in response to high glucose challenge (20 mM glucose concentration) as shown in Figures 5B and 5C.

### BM lowers the levels of extracellular ATP in human islets

We evaluated extracellular ATP accumulation in human islet culture with and without BM as ATP levels act as signals for cell death (Extracellular ATP causes apoptosis and necrosis of cultured mesangial cells via P2X7R). Detectable levels for extracellular ATP in medium range from 1E-16 to 5E+8 as per manufacture instruction. As shown in Figure 5D, ATP accumulation was detected at the beginning of islet-only culture (Day 8), with continuous increases evident on Day 35 and Day 63, whereas ATP in BM-cultured islets was not within the detectable range until Day 63. High-glucose challenge increases ATP accumulation in both islet-only culture and islets with BM culture on Day 8 (Figure 5E). However, BM induced reduction of ATP was observed in high-glucose challenge conditions.

### P2X7 receptor expression

**Figure 6:**
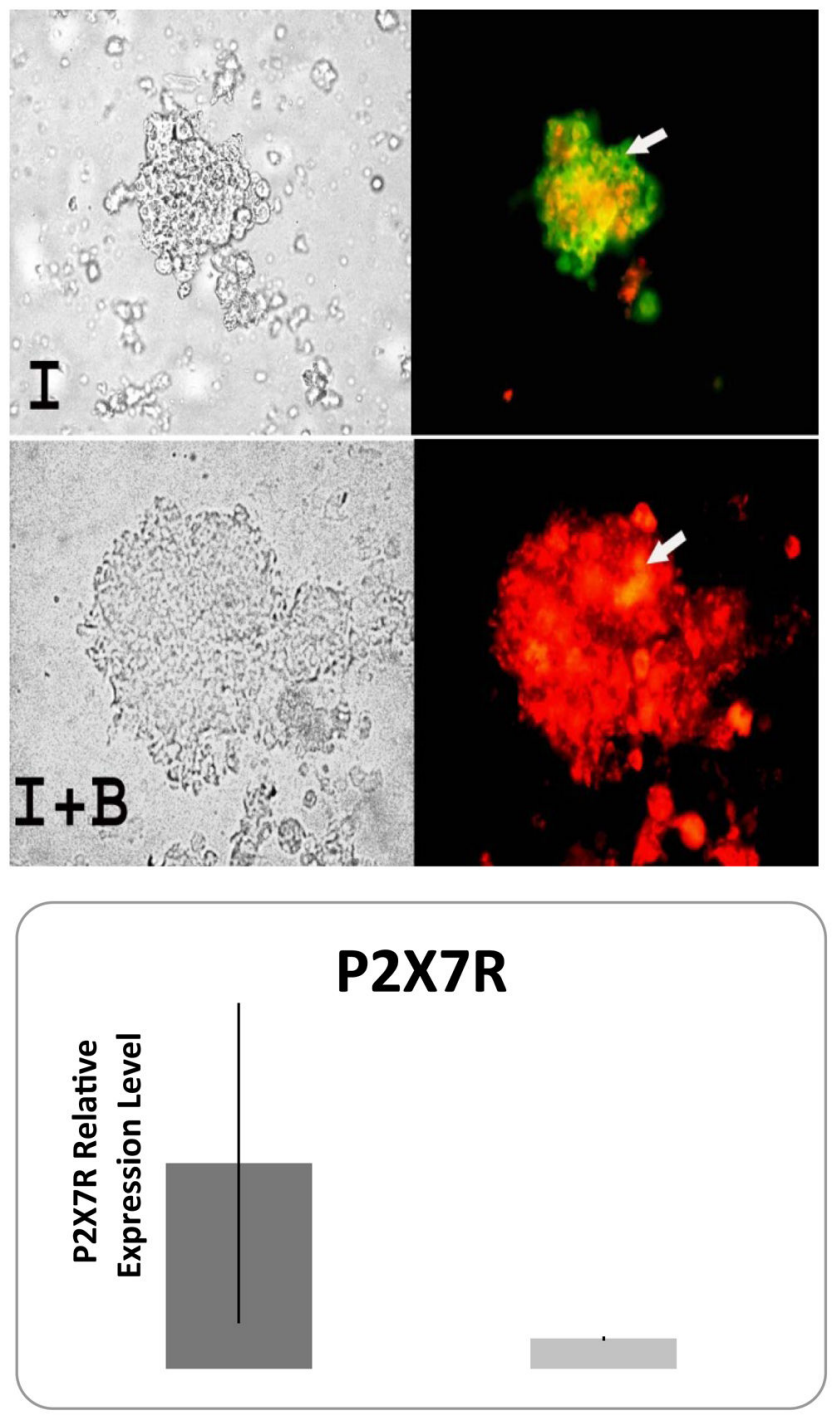
A) Fluorescent Immunohistochemistry staining of P2X7 expression in the human islet. P2X7 was labeled with anti-P2X7 antibody (green, arrow indicated), while β cells were labeled with anti-human proinsulin antibody (red, arrow indicated). Magnification=20X. B) The relative expression level of P2X7R in oxATP treated human islet and human islet control. (*p<0.05 vs. islet human islet control group).

P2XR is ATP-regulated ion channels. In order to identify whether extracellular ATP and its receptors play a role in BM improving islet function, P2X7R expression in islet in both culture systems was evaluated by FIHC. P2X7R in human islets after 29 days of culture was stained (green) and proinsulin was labeled (red) indicating insulin-producing cells within human islets. The negative control with P2X receptor peptide corresponding to P2X receptor antibody showed no signal (image not shown). The islet-only culture showed smaller islet sizes, high expression of P2X receptor (green) and less insulin staining, whereas BM-cultured islets showed opposing results (Figure 6A). In Figure 6B, the q-PCR analysis showed that the expression level of P2X7R can be modulated by adding the antagonist of ATP receptor which could significantly reduce the expression of this receptor. We suspect that bone marrow cells have the same function as the antagonist of ATP receptor.

## Discussion

Human islet apoptosis can be attributed by many factors. The identification human islet apoptosis after 7 hours of culture demonstrated that human islet is very sensitive to a change in environment. The detection of inflammatory factors in accordance with the occurrence of apoptosis showed that human islet apoptosis is inflammation related. The direct interaction between pancreatic β-cells and macrophages, T-cells, exposure to cytokines, as well as other stress inducing molecules triggers β-cell apoptosis. IL-1β is considered one of the most important proinflammatory cytokines triggering the apoptosis cascade through activating cellular COX-2, NF-kb and Fas apoptotic cascades [27]. In the present study, IL-1 β levels in islet only and islets with BM culture was compared. Results indicated null levels of IL-1β in both of islet without or with BM culture in the first 2 weeks, which was consistent with other studies [28]. However, after two weeks the islet-only culture showed an increase in IL-1β (Figure 5B) release under high glucose stimulation for 30 minutes (Figure 5C), which was also consistent with other reports [29–31]. No significant release was detected from BM co-cultured islets in either basal or glucose stimulation conditions, suggesting that BM inhibits IL-1β release, which may play a critical role in promoting human islet survival in vitro. The correlation of human islet insulin concentration with the IL-1β release (Figure 5A) demonstrated that BM maintained human islet function (insulin release) through inhibiting release of IL-1β.

The P2X7R for ATP is expressed predominantly in immune cells and functions as a non-selective ligand- gated ion channel. The activation of purinergic receptors has been identified as an initiator of cellular IL-1β synthesis and release [32,33]. Purinoreceptors have been classified according to their sensitivity to structural analogues of purines. The investigation of the nucleotide receptors P2X1, P2X2, P2X7, P2Y1, P2Y2 and P2Y4 in the pancreas of the streptozotocin (STZ)-induced diabetic rat found that P2X7R expression, normally located in the outer periphery of the islet, was increased inside islets after STZ treatment. Double-labeling experiments showed that an increase in immunostaining for P2X7R and P2Y receptors was present in all pancreatic cell subtypes [34]. Among agents reported to activate purinoreceptors and thus promote cytokine release, ATP, which acts on the ionotropic P2X7R in high concentrations, is one of the most powerful stimuli for the processing and release of IL-1β Large amounts of ATP, accumulated extracellularly at the site of lesion, can directly activate P2X7 mediated IL-1β release, thus activating caspase-1, which initiates the apoptotic cascade leading to cell death [35]. A clinical diabetes study suggests that extracellular ATP regulates several cellular functions via specific purinergic receptors, such as P2X7R [34]. The investigation of glucose uptake in response to purinergic receptor stimulation in fibroblasts from type 2 diabetic (T2D) patients found that extracellular ATP plays a role in the modulation of glucose transport via the purinergic receptor-dependent activation. The high ATP and increasing amounts of P2X7R expression were observed in deteriorating human islets in our study. This is consistent with findings in other pathophysiological conditions. For example, increased extracellular ATP functions as a cytotoxic factor respond for neuron death and pro-inflammatory mediator through P2X7R in neuron disease [36]. The peritraumatic zone in spinal cord injury was characterized by a sustained process of pathologic, high ATP release, and P2X7R expression, which directly caused an increase in cytosolic calcium and cell death [37]. However by adding the P2X7R antagonists, cell apoptosis was prevented [37]. In our study, bone marrow cells blocked the initiation of cell apoptosis by inhibiting P2X7R activation, protecting human islet β-cell from apoptosis (Figure 6). Furthermore, islet function in terms of insulin release was maintained in comparison with the deterioration occurred in islet only group (Figure 5A), which is consistent with our previous studies [38]. The correlation of insulin and IL-1β release demonstrated that the reduction of insulin concentration might be related with the increasing release of IL-1β in islet only cultures, while BM co-culture prevented the increase in IL-1β. This finding confirmed previous studies that inflammatory cytokines inhibited insulin secretion in human islet [16]. The usage of P2X7 antagonist ox-ATP in our study showed that human islet function can be enhanced by the treatment of ox-ATP. These results suggest that reduction of extracellular ATP or the inhibition of P2X7R expression could be an additional therapeutic target for improving glucose utilization in diabetic conditions [39].

Although oxidation of extracellular ATP and reduction of ATP accumulation may benefit islet survival, this issue has not yet been investigated. Evidence that hypoxia reduces organ preservation and islet yield has been well known [40]. The absence of oxygen halts the degradation of extracellular ATP leading to the increase of ATP accumulation and possibly results in islet cell death *in vitro*. The accumulation of extracellular ATP in damaged islets results in the activation of P2XR and triggers IL-1β-induced cell death. Several studies have found that members of the MAP kinases family, including the ERK, JNK, and p38 kinases contribute to the regulation of islet cell apoptosis and regeneration [41–44]. Further investigation of these mechanisms may open a new avenue of research on the reduction of islet loss *in vitro*.

Although significant achievements have been made in the differentiation of insulin-producing cells from embryonic and adult stem cells [45], the loss of long term islet function and viability has yet to be fully addressed. When mice subjected to islet damage were injected with labeled BM cells, BM cells were not shown to develop into insulin producing cells. However, there were many BM derived endothelial cells present in the areas of injury. The data therefore indicate that BM derived endothelial progenitor cells respond to cell injury in the pancreas and that this mechanism might be beneficial for the repair islet damage to improve islet survival [46]. BM involvement in pancreatic islet development during the neonatal period and after pancreatic injury also supports this possibility [47,48].

An intervention which reduces or prevents islet immunoreactions resulting in apoptosis or necrosis is crucial in preventing islet loss during transplantation [49]. We believe that human islet damage sustained during the isolation process might be repaired by allogeneic BM and that BM can be hold multiple functions acting as a scaffold material, which may stimulate islet regeneration into a 3D structure. Our preliminary studies suggest that BM has the ability to release cytokines (paracrine) to promote islet survival and restrict the extracellular adenosine triphosphate (ATP) accumulation and modulated miR146a that results in cell death or damage, deactivate purinoreceptor (P2XR) expression in turn to limit the release of inflammatory factors, such as interleukin 1β (IL-1β), thus preventing non-specific inflammatory response and reducing cell death.

## Conclusion

In summary, bone marrow cells can significantly reduce apoptosis in human islet cells. We believe one of the mechanisms is related to the suppression of inflammation, specifically by reducing the levels of ATP and its receptors P2X7 expression, thus inhibiting the release of inflammatory factor IL-1β. Bone marrow cells can be used as a functional tool for sustaining human islets function and survival.
